# Pharmacokinetics of anticoagulant edoxaban in overdose in a Japanese patient transported to hospital

**DOI:** 10.1186/s40780-020-00176-6

**Published:** 2020-09-11

**Authors:** Koichiro Adachi, Jumpei Tuchiya, Satoru Beppu, Kei Nishiyama, Makiko Shimizu, Hiroshi Yamazaki

**Affiliations:** 1 Laboratory of Drug Metabolism and Pharmacokinetics, Showa Pharmaceutical University, 3-3165 Higashi-tamagawa Gakuen, Machida, Tokyo 194-8543 Japan; 2 Kyoto Medical Center, Fushimi-ku, Kyoto, 612-8555 Japan

**Keywords:** Anticoagulants, Pharmacokinetic modeling, Overdose

## Abstract

**Background:**

The anticoagulant edoxaban is used clinically at doses of 30–60 mg/day; however, we experienced a patient who had taken an overdose of edoxaban of 750 mg. We investigated the pharmacokinetics of edoxaban in this patient by using liquid chromatography–tandem spectrometry to estimate the follow-up period in emergency clinical practice with this medicine.

**Case presentation:**

The patient was a 57-year-old woman (body weight, 69 kg) who had taken a single oral dose of 750 mg of edoxaban in a suicide attempt. She was emergently admitted to Kyoto Medical Center. The patient’s edoxaban plasma concentrations during ambulance transport (8 h after oral administration) were ~ 4900 ng/ml, and the concentration gradually decreased to ~ 10 ng/mL and to detectable but unmeasurable levels of ~ 1.0 ng/mL at 60 h and 100 h, respectively. The linear range of the relationship between the dose and plasma concentration was assumed to have been exceeded during the first 8 h; however, the measured elimination rate of edoxaban was similar to that visualized curves predicted by a simplified physiologically based pharmacokinetic model previously established.

**Conclusion:**

Simplified physiologically based pharmacokinetic models for creating visualized curves have proven to be useful not only during drug discovery or chemical risk assessment but also in cases of medical poisoning. We used a physiologically based pharmacokinetic model previously established for edoxaban to predict the pharmacokinetics in the current case. It is hoped that the results of this study, which encompass drug monitoring data in the patient and visualized pharmacokinetic prediction, will serve as an index when setting the treatment and follow-up period in cases of drug overdose for medicines such as edoxaban in emergency clinical practice.

## Background

Direct oral anticoagulants have gained increasing popularity for the treatment of venous thromboembolism because of their wide therapeutic ranges and predictable pharmacological effects [1, 2]. After aspirin discontinuation, platelet reactivity increased to a great extent with a low-dose of anticoagulant edoxaban [3]. However, a recent study reported that hospitalizations as a result of bleeding events associated with treatment with direct oral anticoagulants (apixaban, dabigatran, and rivaroxaban) have been increasing [4]. Despite initially not being recommended, the monitoring of plasma concentrations of anticoagulants should now be recognized as essential in emergency situations and in special populations. However, only limited information is available on the pharmacokinetics of direct oral anticoagulants.

Therapeutic drug monitoring is accepted to be the clinical practice of measuring specific and drugs (such as antibiotics) in blood samples from patients at designated intervals to maintain a range of constant drug concentrations. For creating these visualized plasma concentration curves for limited antibiotics, some computer system has been freely supported by the drug manufacturers or commercially provided by software houses. However, many medicines in critical care area have not been fully supported by simple and easy handling software system for visualizing plasma concentration curves in clinical hospital pharmacies [5, 6].

In general, drug monitoring of steady-state plasma concentrations of individual patients in a clinical setting could be predictable by complex and detailed physiologically based pharmacokinetic (PBPK) models consisting of multiple compartments [7]. However, such PBPK models are impractical for widespread daily use in clinical hospital pharmacies to support the care of a verity of patients. Against this background, we proposed simple PBPK models for drugs for practical use by paramedical staff [8]. A prototype PBPK simulator [9] for creating the visualized plasma concentration curves for important medicines by easy input was provided by our group and has been currently updated and modified. Simple pharmacokinetic models and simulation systems would be expected for the drug monitoring results even in emergency rooms.

## Case presentation

Here we describe the case of a woman who intentionally took an overdose of edoxaban (usual clinical dose in the range 30–60 mg/day). The patient’s plasma concentrations during ambulance transport (~ 4900 ng/ml) after an oral overdose of 750 mg of edoxaban gradually decreased to ~ 370 ng/mL at 28 h after administration. The linear range for doses and plasma concentrations was assumed to have been exceeded during the first 8 h because of saturated elimination. However, the predicted plasma concentration curve showed a linear relationship. We report herein the drug monitoring data and the results of pharmacokinetic modeling, which indicate that such prediction is a suitable tool for application in an emergency. The ethics committee of Kyoto Medical Center approved this study.

The patient was a 57-year-old woman (body weight, 69 kg) who had taken a single oral dose of 750 mg of edoxaban as a suicide attempt. She was emergently admitted to Kyoto Medical Center. The patient was then administered vitamin K (20 mg) as primary treatment. To assess the effects of treatment, plasma samples were collected from the patient at seven time points (8, 28, 36, 52, 60, 76, and 100 h after oral administration) and were frozen. The patient gave written informed consent to take part in this study and for publication. Concentrations of edoxaban in the plasma samples were analyzed using liquid chromatography–tandem mass spectrometry assays according to previously described methods [10]. Under the present conditions, edoxaban in plasma was measurable (≥ 2.0 ng/mL) or detectable (≥ 0.20 ng/mL) and each time point.

A simplified PBPK model consisting of gut, liver, and central compartments was previously set up for patients receiving normal clinical oral doses of edoxaban [10]. This model was employed in the current study without making changes to the input parameters, such as the physiological hepatic blood flow rate (96.6 L/h), the hepatic volume (1.5 L), the urinary volume (1.5 L/day), and the body weight (70 kg). Calculated parameters for the PBPK model for absorption rate constant, fraction absorbed × intestinal availability, volume of systemic circulation, hepatic intrinsic clearance, hepatic clearance, and renal clearance were 1.33 1/h, 0.700, 127 L, 30.3 L/h, 40.2 L/h, and 10.0 L/h, respectively, as described previously [10]. The 95% confidence intervals (CIs) were estimated for the fitted intrinsic hepatic clearance values using 100 virtual subjects created using random numbers, as described previously [10]. The mean hepatic clearance in the previous PBPK model was approximately 2-fold to a total clearance of 21.8 L/h for edoxaban (shown in the package insert of edoxaban in Japan). The predicted human virtual output C_max_ and AUC_0–24_ values extrapolated from the previous PBPK model were consistent with those taken from reported means in subjects, supporting the relatively good fitting results (within roughly a two-fold range of predictions) as described previously [10].

The clinical laboratory results for the patient who had taken a single oral dose of 750 mg of edoxaban are shown in Table 1. Prolonged activated partial thromboplastin times and prothrombin times, as markers for blood coagulation disorder, were observed from the beginning of hospitalization to 13 h after the oral dose; no bleeding occurred during this time. These parameter values had decreased to the normal levels at 28 h, and these normal levels were maintained up to 60 h after oral doses. Other laboratory test results for hemoglobin, hematocrit, and platelet count were essentially stable during the 60-h observation period. It was assumed that apparent normal renal function of the subject and possible little interactions with concomitant drugs shown in Table 1 would not cause any saturation of edoxaban clearance.

**Table 1:** Clinical laboratory results in a patient who had taken a single oral overdose of 750 mg of edoxaban

	Time after administration (h) of oral dose					
	8	13	28	36	52	60
Activated partial thromboplastin time (s)	59.4	43.0	30.9	26.2	26.1	25.9
Prothrombin time (s)	54.8	33.7	14.9	13.4	11.5	11.5
International normalized ratio	4.34	2.69	1.21	1.09	0.94	0.94
Hemoglobin (g/dL)	12.8		12.1		11.9	12.1
Hematocrit (%)	36.2		35.2		36.4	33.4
Platelet count (per μL)	197,000		168,000		187,000	170,000
Serum creatinine (mg/dL)	0.76	0.82	0.93	0.82	0.83	0.73
Creatinine clearance (mL/min)	89	82	73	82	81	93

Concomitant drugs for this subject were fluvoxamine, lorazepam, valproic acid, promethazine, quazepam, levomepromazine, sertraline, quetiapine, esomeprazole, and polycarbophil calcium, which might not substantially affect the edoxaban pharmacokinetics

## Discussion and conclusions

**Fig. 1:**
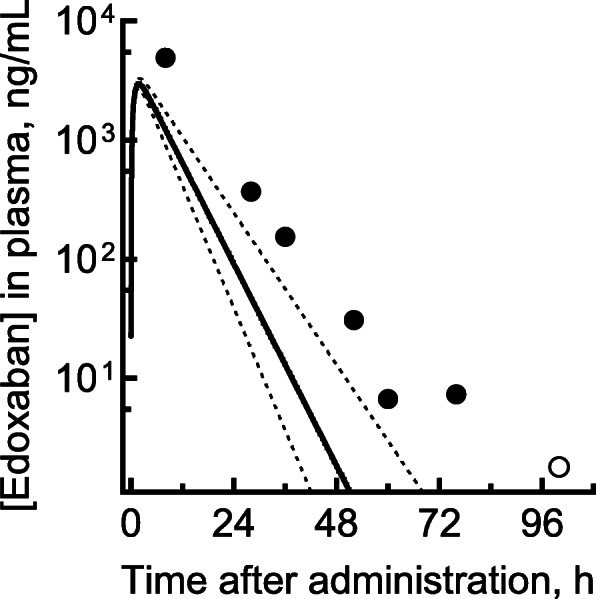
Measured (circles) and estimated (lines) plasma concentrations of edoxaban in a patient who took an excessive single oral dose of 750 mg. The plasma concentration curve after virtual administration of 750 mg of edoxaban (solid line) is shown with the 95% confidence interval (broken lines) based on the hepatic intrinsic clearance values of 100 virtual subjects created using random numbers, as described previously [10]. The open circle at 100 h indicates that edoxaban was detectable, but below the measurable limit, using the present liquid chromatography–tandem mass spectrometry system

The measured plasma concentrations of edoxaban after the single oral overdose are shown in Fig. 1. The plasma concentration of edoxaban at 8 h after administration was 4920 ng/mL, and the concentrations gradually decreased to 368 ng/mL, 154 ng/mL, and 31 ng/mL at 28, 36, and 52 h after administration. The plasma concentration was still at a measurable level of around 7 ng/mL even 60 and 76 h after administration. Edoxaban in plasma was detectable (~ 1.8 ng/mL), but was judged to be unmeasurable (i.e., below the measurable level of 2.0 ng/mL), 100 h after administration.

Predicted virtual plasma concentration curves (with 95% CIs based on the variation in hepatic intrinsic clearance values) are plotted along with the measured plasma concentrations of edoxaban versus time after the overdose administration (Fig. 1). The observed concentrations in the patient who took an overdose of 750 mg edoxaban were higher than the 95% CI of the predicted plasma concentration curves. The measured AUC_0–100_ was calculated to be 75.9 μg・h/L, which was 3-fold to the predicted AUC_0–100_ (24.6 μg・h/L) in the linear model. However, the disappearance rate of edoxaban, even for such a large overdose, generally matched the slope predicted by the PBPK model. It was assumed that the linear range was exceeded at least up to 8 h after oral administration, presumably because of metabolic saturation during the process. A lesson from this overdosed case was that later phase of edoxaban would be within the linear range, but initial phase might be out of range of linearity probably because of saturated elimination caused by the overdose. Visualized pharmacokinetic prediction curves by simple simulators will serve as an index when setting the treatment and follow-up period in the future cases of drug overdose for medicines such as edoxaban in emergency clinical practice. These observations could help in understanding the predictive pharmacokinetics of edoxaban, in setting the follow-up, and in determining whether to administer gastric lavage and/or blood products in the patients.

User-friendly bioanalytical techniques are still required to predict plasma concentrations of edoxaban in routine clinical practice and in phase IV clinical trials ongoing worldwide. At present, no drug that selectively neutralizes the anticoagulant effects of edoxaban has been marketed in Japan. According to patient interview forms, edoxaban is difficult to remove by hemodialysis. Therefore, the administration of concentrated red blood cells or fresh frozen plasma transfusion are considered as symptomatic treatments in patients whose plasma concentrations of oral thrombin or factor Xa inhibitors have become dangerously high [11]. In the current patient, blood coagulation disorder was observed; however, the patient was followed up for 100 h, because of the possibility of asymptomatic effects. Because the pharmacokinetics of edoxaban overdose were unknown, a clear follow-up period has not been set for emergency clinical practice.

In the present study, by using drug monitoring data and the results of pharmacokinetic prediction, we revealed the pharmacokinetics of edoxaban in a patient who took an overdose. It was evident that the PBPK model constructed as part of a previous study based on normal clinical oral doses of edoxaban [10] without any making changes with visualized blood concentration curves of drugs could be applied successfully in the clinical setting. Application of the PBPK model simulators may help in understanding the pharmacokinetics of edoxaban, in setting the follow-up period, and in determining whether to administer blood products and/or gastric lavage. Furthermore, this type of PBPK model simulator may be applicable for patients who have taken overdoses of other drugs.

The simplified PBPK model applied here is useful not only in the drug discovery or chemical risk assessment field but also in the management of poisoning. The simplified model simulator has the advantage that it can be handled by clinicians who are not experts in pharmacokinetics. Especially, in hospitals without systems for measuring blood levels of drugs routinely, the simplified PBPK model simulator should be of use. It is hoped that the results of this study with drug monitoring data and pharmacokinetic prediction will serve as an index when setting the treatment period in cases of overdoses of drugs such as edoxaban.

## Data Availability

All data generated or analyzed during this study are included in this published article and are also available from the corresponding author on reasonable request.

## Abbreviations

CIs: Confidence intervals
PBPK: Physiologically based pharmacokinetic
